# Ultrasound’s ‘window on the womb’ brings ethical challenges for balancing maternal and fetal health interests: obstetricians’ experiences in Australia

**DOI:** 10.1186/s12910-015-0023-y

**Published:** 2015-05-08

**Authors:** Kristina Edvardsson, Rhonda Small, Ann Lalos, Margareta Persson, Ingrid Mogren

**Affiliations:** Department of Clinical Sciences, Obstetrics and Gynecology, Umeå University, SE 901 87 Umeå, Sweden; Judith Lumley Centre, La Trobe University, Melbourne, Vic 3000 Australia; Department of Nursing, Umeå University, SE 90187 Umeå, Sweden

**Keywords:** Australia, Decision-making, Ethics, Fetus, Maternal rights, Obstetric ultrasound, Obstetricians, Obstetrics, Pregnant women, Qualitative studies

## Abstract

**Background:**

Obstetric ultrasound has become a significant tool in obstetric practice, however, it has been argued that its increasing use may have adverse implications for women’s reproductive freedom. This study aimed to explore Australian obstetricians’ experiences and views of the use of obstetric ultrasound both in relation to clinical management of complicated pregnancy, and in situations where maternal and fetal health interests conflict.

**Methods:**

A qualitative study was undertaken as part of the CROss-Country Ultrasound Study (CROCUS). Interviews were held in November 2012 with 14 obstetricians working in obstetric care in Victoria, Australia. Data were analysed using qualitative content analysis.

**Results:**

One overall theme emerged from the analyses: *The ethical challenge* of *balancing maternal and fetal health interests,* built on four categories: First, *Encountering maternal altruism’* described how pregnant women’s often ‘altruistic’ position in relation to the health and wellbeing of the fetus could create ethical challenges in obstetric management, particularly with an increasing imbalance between fetal benefits and maternal harms. Second, *‘Facing shifting attitudes due to visualisation and medico-technical advances’* illuminated views that ultrasound and other advances in care have contributed to a shift in what weight to give maternal versus fetal welfare, with increasing attention directed to the fetus. Third, *‘Guiding expectant parents in decision-making’* described the difficult task of facilitating informed decision-making in situations where maternal and fetal health interests were not aligned, or in situations characterised by uncertainty. Fourth, ‘*Separating private from professional views*’ illuminated divergent views on when the fetus can be regarded as a person. The narratives indicated that the fetus acquired more consideration in decision-making the further the gestation progressed. However, there was universal agreement that obstetricians could never act on fetal grounds without the pregnant woman’s consent.

**Conclusions:**

This study suggests that medico-technical advances such as ultrasound have set the scene for increasing ethical dilemmas in obstetric practice. The obstetricians interviewed had experienced a shift in previously accepted views about what weight to give maternal versus fetal welfare. As fetal diagnostics and treatment continue to advance, how best to protect pregnant women’s right to autonomy requires careful consideration and further investigation.

## Background

Obstetric ultrasound has become a significant tool in obstetric practice worldwide. It reached widespread use in developed countries in the 1990s, and today, women in most developed countries are offered at least one ultrasound in uncomplicated pregnancies, in some countries up to four [1]. The use of obstetric ultrasound is also fast becoming ingrained in obstetric care in developing countries [2,3].

The main benefits of ultrasound include early detection of multiple pregnancies, localization of the placenta, estimation of gestational age [4] and detection of fetal anomalies [5]. The use of ultrasound has also been shown to reduce the risk of perinatal death and the use of obstetric interventions in high risk pregnancies [6]. However, there is to date no supporting evidence that routine scans in early or late pregnancy confer benefits for mothers or babies if used in low-risk or unselected populations [4,7]. Obstetric ultrasound examinations are popular among expectant parents [8,9], and the vast majority of women participate in the routine scans offered during pregnancy [10,11]. The routine scan is often experienced as a significant event where the pregnant woman and her partner are given the opportunity to have a ‘sneak peak’ at their future baby and to obtain the first image for the family album [8,9]. Most women expect to be reassured that everything is fine with the pregnancy during this examination [9,11]. At the same time, women often lack information about the purpose of routine scans [9], which can make unexpected findings even more distressing and difficult to deal with [9,12].

There has been discussion about the introduction and increasing use of obstetric ultrasound as resulting in a change in the perception of the fetus [13]. Enhanced visualisation and improved imaging technology, particularly 3-D and real-time 3-D, has led to increased ‘personification’ of the fetus. Likewise, the fetus has come to acquire a social identity by its public presence [14], a social status that before the introduction of imaging technologies was not acquired until after birth [13]. Modern medical capacity to visualise the fetus in the uterus, combined with increased capacity for fetal treatment, have also led health professionals conceptualising the fetus as a patient [15,16]. Moreover, it has contributed to individual rights being increasingly attributed to the fetus [17], and to some extent the woman has lost her central place in the pregnancy [13,17]. It is a human right to consent to or refuse medical treatment or interventions and everyone has the right to make their own decisions about their bodies [18]. Pregnant women are no exception. Women’s right to decline obstetric interventions can however, give rise to complex ethical dilemmas in obstetric care [19].

It has been argued that the increasing use of visualisation technology may have adverse implications for women’s reproductive freedom [17]. To date however, this issue has not been sufficiently studied in different social, cultural and economic contexts, nor satisfactorily discussed. The present study is part of the CROss-Country Ultrasound Study (CROCUS), which has an overall purpose to explore issues related to the use of obstetric ultrasound in low-income and high-income countries [20]. The specific purpose of this sub-study was to explore obstetricians’ views and experiences of the use of obstetric ultrasound both in relation to clinical management of complicated pregnancy, and in situations where maternal and fetal health interests conflict.

## Methods

### Study design

A qualitative study design was applied, involving interviews with obstetricians working in obstetric practice. Transcribed data underwent qualitative content analysis.

### Participants

Fourteen obstetricians were recruited from two large hospitals in Victoria, Australia, each with more than 4000 births per year. The recruitment of participants was undertaken with assistance of the department heads. The inclusion criteria were being an obstetrician working mainly with obstetric ultrasound, working with ultrasound as part of obstetric care, or using the results of obstetric ultrasound in clinical management of pregnant women even if not performing obstetric ultrasound examinations themselves. Ten of the participants were females and four were males. The mean age was 43.7 years (range 33 - 59 years), and the participants had varying experience in obstetrics, with a mean of 15.5 years (range 4 - 30 years). A few were of non-Australian origin and had obstetric experience from other countries. All were qualified in performing obstetric ultrasound examinations. According to information provided by department heads, all obstetricians they approached agreed to participate.

### Data collection procedures

An interview guide with key domains linked to the overall aims of the CROCUS study was developed by the research team (Table 1). Interviews took place at the two hospitals in November 2012 and were performed individually by two of the authors (IM and MP) during the obstetricians’ ordinary work shifts. The use of an interview guide ensured that all topics were covered in each interview; however, the questions were not asked in a predefined order, which meant that the participants’ narratives were not interrupted. The interviews lasted on average 37 minutes (range 22 - 65 minutes), were digitally recorded and later transcribed verbatim. Demographic information was collected at the time for the interview via a short anonymous questionnaire. The key areas of focus for this paper were: clinical situations where maternal and fetal health interests were in conflict; whether the woman may be considered to act as an instrument for fetal treatment; if or when the fetus can be regarded as a person; situations where the fetus has been regarded as a patient with his/her own interests; and other issues in relation to ethical aspects of the use of obstetric ultrasound. Other results have been published elsewhere [20].

**Table 1:** Key domains in the interview guide linked to the overall aims of the CROCUS study*

**Key domains in the CROCUS study**	The obstetricians’ experiences and views of:
	• The importance/value of obstetric ultrasound for clinical management of complicated pregnancy.
	• **Clinical situations where the interests of maternal and fetal health have been in conflict.**
	• **Whether the woman may be considered to act as an instrument for fetal treatment.**
	• The importance of obstetric ultrasound in comparison to other surveillance methods during complicated pregnancy.
	• **If/when the fetus can be regarded as a person.**
	• **Situations where the fetus has been regarded as a patient with his/her own interests.**
	• Their professional role in relation to other occupational groups working with obstetric ultrasound examinations or the outcomes of these examinations.
	• **Other issues in relation ethical aspects of the use of obstetric ultrasound.**

*Key domains addressed in this sub-study are indicated in bold text.

### Data analysis

Data were analysed using qualitative content analysis [21]. First, the transcribed interviews were read by three of the authors for familiarization with the data (KE, RS and IM). Recurrent topics were identified during this process and key ideas discussed. KE then analytically coded all data, and IM and RS coded parts of the data to check consistency of coding. Codes with similar meaning or content were brought together in broad content areas, which were then further refined by the development of categories and an overall theme. The analysis, performed by KE in close collaboration with IM, involved an iterative process, i.e. constantly moving back and forth between the original text, codes, categories and an emerging theme, in order to represent fully the underlying meanings in the data [21]. All authors reviewed the preliminary results, and remaining uncertainties in interpretation and labelling of categories and the theme were resolved through joint discussion.

### Ethical considerations

Verbal and written informed consent was obtained prior to the start of each interview, and all participation was voluntary. Ethics approval was obtained from the Faculty Human Ethics Committee at La Trobe University in Melbourne (reference FHEC12/135) and the Human Ethics Committees of the two participating hospitals prior to data collection.

## Results

### The ethical challenge of balancing maternal and fetal health interests

One overall theme emerged from analysis: ‘The ethical challenge of balancing maternal and fetal health interests’. This theme was built on four categories that describe different aspects of the challenges obstetricians faced in management of complicated pregnancies, including the balancing of maternal health interests and fetal health interests, as is inevitably required in obstetric practice. Each category is presented below and quotations are used throughout the text to illustrate how the interpretation is grounded in the data. An overview of the theme and categories is presented in Table 2.

**Table 2:** **Theme and categories**

**Theme**	**The ethical challenge of balancing maternal and fetal health interests**			
Categories	**I. Encountering maternal altruism**	**II: Facing shifting attitudes due to visualisation and medico-technical advances**	**III. Guiding expectant parents in decision-making**	**IV. Separating private from professional views**

*‘I guess always in obstetrics it’s a balance to be struck between the welfare of the mother and the welfare of the baby.’* (Participant no 8)

*‘Trying to balance those fetal and maternal … yeah priorities I think is very, very difficult.’* (Participant no 1)

### I. Encountering maternal altruism

The pregnant woman’s ‘altruistic’ position in relation to the health and wellbeing of the fetus was a common topic that emerged during interviews. The obstetricians commented that almost with no exception, women would go through a great deal and put up with inconvenience, discomfort, pain and even risks to their own health in order to protect the health of the fetus. However, clearly discernible in the narratives was a perception of greater ethical conflict in relation to the increasing imbalance between maternal and fetal harms and benefits, situations interpreted as ultimately resulting from continuing advances in fetal diagnostics and treatment.

The first and most common situation described, but also the least problematic to manage from an ethical perspective, was when pregnant women experienced inconvenience, discomfort and possible medical risks, but at the same time there was a good deal of confidence that this maternal sacrifice would pay off in terms of positive fetal health outcome. According to the obstetricians, tough treatments were commonly not seen by pregnant women as too difficult if considered likely to be effective. Examples given included situations where pregnant women underwent intrauterine fetal blood transfusions, received substantial treatment of fetal arrhythmia, underwent caesarean section on fetal indication, or endured bed rest for long periods in the hope of prolonging a threatening preterm pregnancy.

A somewhat more difficult ethical conflict was said to occur when pregnant women were prepared to make choices to secure the health of the fetus or to improve fetal outcome that would most likely cause morbidity for themselves. The obstetricians described situations where pregnant women literally said they would sacrifice themselves for the fetus or the health of their baby, and they reported that some women would not listen to information about the potential adverse health consequences of their decisions. This was said to be particularly common among pregnant women who knew they would have limited opportunities to have another pregnancy. One example of such a difficult maternal-fetal health conflict was when women developed severe pre-eclampsia around the time of fetal viability. These situations were described as challenging for the expectant parents as well as for caregivers.

*‘Some of the time they just say: do whatever you can for the baby, and they really don’t listen to you saying there are these downsides.’* (Participant no 12)

*‘A classic example would be if the mother has severe pre-eclampsia at 24 weeks of pregnancy… The baby might be growth restricted, barely viable, and the likelihood of the mother being able to continue the pregnancy before harm befalls her of a significant nature is minimal… the advice that I think should be given is that the pregnancy be terminated and the fetus sacrificed for the welfare of the mother.’* (Participant no 11)

It was not only where a good fetal outcome was expected that obstetricians described pregnant women putting themselves at medical risk. Sometimes a woman insisted on interventions that would entail a medical risk for her, even though it was not expected to make any difference to fetal health or survival. The obstetricians experienced some pregnant women wanting to persist with the pregnancy beyond the point where they knew their health would be at significant risk. When the fetus was unlikely to survive, but where the mother insisted on prioritising her ‘baby’, some obstetricians thought that it was their role to say stop. They felt it was incumbent on the treating team to ensure that adverse effects or disadvantages were proportional to the expected benefits.

*‘The problem starts when you have a really sick baby and you know that you’re going to do a big operation for a baby that might not survive. I do have problems with those.’* (Participant no 5)

*‘There is that maternal selflessness so we may need to contain it, you know, in some ways saying no, this is too much, you know?’* (Participant no 13)

### II. Facing shifting attitudes due to visualisation and medico-technical advances

The obstetricians all agreed that visualisation through ultrasound and the technique’s immediacy were invaluable in the management of pregnancy. However, they discussed how ultrasound has come to influence the balancing of maternal and fetal well-being, both in obstetric care and in society in general. Some obstetricians reported experiencing a shift in attitude to the woman’s role during pregnancy, partly because ultrasound provided a ‘window on the fetus’ where the fetus and its world is now on view for the world outside the womb.

Ultrasound was seen by the obstetricians as valuable in helping couples bond with the fetus during pregnancy. However, they discussed the implications of visualisation in circumstances where there was a conflict between maternal and fetal health interests. The visualisation was described as generating a greater emotional attachment to the fetus both for the woman and her partner and at times even for the doctor caring for her, so that decision-making about what course of action to take in situations of conflicting health interests could be very emotionally charged.

*‘I think that view is one that’s been traditionally the case in obstetrics that the mother always takes precedence over the fetus but increasingly with the window on the fetus and its world that’s provided by ultrasound there is a shift in the attitude.’* (Participant no 11)

It was not only ultrasound in isolation, but medico-technical advances in obstetrics overall, that were seen as contributing to shifting attitudes in relation to the balance between maternal and fetal well-being. Some obstetricians suggested that because of new diagnostic and treatment possibilities, the perspective of always favouring the health of the pregnant woman over the health of the fetus was not as clear and straightforward today as earlier in their careers. For example, the parallel medico-technical advances in neonatal care was said to influence obstetricians’ willingness to intervene on behalf of the fetus. Furthermore, some presented the view that advances in reproductive technology and genetics had also influenced what weight to give maternal welfare versus fetal welfare. A few obstetricians described how they had experienced situations in practice, where, although rare, the focus had shifted from the pregnant woman to the fetus. Some of these experiences were in the context of highly specialised health care.

*‘Advances in neonatal care, and that involves not only say corticosteroids for fetal lung maturation but also magnesium therapy for fetal neuro protection. These sorts of advancements, which have good evidence behind them are, I think, influencing our views as to what weight to give to fetal welfare versus maternal welfare.’* (Participant no 11)

Developments in the area of fetal medicine were seen as particularly influential in terms of shifting attitudes. Some obstetricians reported situations where there was a sense that the pregnant woman was ‘in the way’, or hindering what doctors might be able to do for the fetus. Examples included fetal surgery for certain congenital malformations. One obstetrician pictured an extreme of this situation in the following way:

*‘I’m not certain if I … this is a caricature, it’s a very extreme example, it seemed to me that barrier of maternal skin simply became that, it was the barrier between them and the fetus, that they somehow needed to breach that barrier to get at the baby and it became like that, just because they could do it and all they wanted was to get a global agreement from the woman, tell us and we will reach the baby and we will do what we need to do, it became like that sometimes, okay, simply because we had the technology to do it.’* (Participant no 8).

Some obstetricians acknowledged that the reason for performing interventions was sometimes that the technology was there, and there was an excitement over new possibilities, rather than because the interventions were well justified.

*‘And that is the danger, that it became very much a process, what next, what can we do next, what can we do next, rather than pull back and say let’s just stop and look at things now, is this too much, is it going to change anything for the baby or are you just doing it because you are keeping yourselves busy actually and sometimes just because the mother says do it doesn’t mean that we should do it.’* (Participant no 8)

Also raised was the potential risk of pregnant women putting fetal health interests ahead of their own in relation to medico-technical advances. Some saw the potential risk in ‘taking advantage’ of this situation for some doctors who had fetal interests at heart or who were keen on using available techniques or treatments. The participating obstetricians saw it as their responsibility however to ensure that interventions were justifiable, especially in light of the fact that most women would do ‘anything’ and sacrifice their own health for the benefit of the fetus.

### III. Guiding expectant parents in decision-making

The obstetricians emphasised the importance of providing expectant parents with the best information possible for them to make informed decisions in situations where an ultrasound examination had provided evidence of abnormality, or where maternal and fetal health interests were in conflict. The value of working at a tertiary hospital where all relevant specialists were available was emphasised, and multidisciplinary team work was described as central in such circumstances:

*‘I think we do everything in a very ethical way and very non-directive. We give parents the facts, we provide them with a multidisciplinary assessment with paediatrician, with geneticists, psychiatrists, and they only make a decision after they’ve heard all the specialists, take in all the different shades. That’s when they make a decision.’* (Participant no 10)

Although ultrasound was described as an important tool for decision-making and for planning of care in complicated pregnancy, the results from an ultrasound examination could also make decision-making for expectant parents very difficult at times. This happened particularly in situations where there was uncertainty regarding the significance of the findings and where the expectant parents were faced with probability estimates regarding fetal outcomes. These situations were depicted as sometimes unmanageable for expectant parents, especially for those who did not want to have a disabled child, but at the same time did not want to make the decision to terminate the pregnancy.

The issue of assisting expectant parents to understand the medical complexity of some situations was frequently mentioned. It was evident from the obstetricians’ narratives that it was easier to manage situations of maternal and fetal health conflicts if patients were well informed, and also if the expectant parents were clear about their wishes all through the pregnancy. These examples included expectant parents who for religious reasons would never consider termination even if the ultrasound showed fetal abnormalities.

*‘In the end of the day, if patients are capable of making the decisions and they … most are, almost all of them are, then yeah, you have to go by what they want.’* (Participant no 5)

Factors such as being competent to make decisions for themselves, and not being coerced by someone else, were noted as important. However, the obstetricians described sometimes finding it difficult to communicate relevant information, especially as emotional stress for expectant parents influenced their capacity to make sense of the situation.

Although some obstetricians emphasised shared decision-making, most claimed that in the end, the pregnant woman was free to decide on treatment. They emphasised that they needed to be supportive of women’s wishes as long as there was no major threat to the woman’s own health. However, this was problematised in some of the narratives. First, some claimed it was not a ‘fair choice’ in certain situations because most pregnant women would choose to have anything done for the benefit of the fetus, even though it might entail risks to their own health.

*‘I don’t think it’s an honest decision for parents to make because all parents will say do what’s best for this baby.’* (Participant no 5)

Second, some obstetricians thought it was somewhat unfair to leave the decisions to pregnant women or couples when they might not be fully able to make informed decisions for a range of reasons. They mentioned it was particularly challenging to communicate complex information when the expectant parents had very low levels of education, were non-English speaking, or came from other cultural settings where the technology and treatments used in the Australian obstetric setting were not familiar. The complexity of decision-making in situations where the expectant parents had mental disabilities was also mentioned.

*‘The subtleties of morbidity, mortality statistics, procedural aspects and the ability stepping down from that … the ability for a patient to interpret and understand all aspects of it, emotional, medical, varies from one patient to the next.’* (Participant no 14)

*‘…especially when they come from a lower socioeconomic area or aren’t … you know, sometimes you really need to be quite educated to understand what we’re all saying otherwise … and again that’s what I think sometimes is very unfair that decisions are left with parents that sometimes aren’t really, yeah, decisions that they can’t make. It’s a big thing.’* (Participant no 5)

A few participants touched upon the importance of preventing regret in situations of complex decision-making. Regret after poor fetal outcome, or when women or couples were unhappy later on with the decisions they had made, were described as difficult for obstetricians to manage well.

*‘And it’s difficult because we see regret after a poor outcome, people feeling regret. If only I’d done this or if only I … a lot of those things that they’re regretting are utterly irrelevant to the outcome, it wouldn’t have made any difference. But trying to avoid regret I think is very important.’* (Participant no 1)

However, at the same time, one obstetrician suggested that accepting expectant parents’ right to decide and entrusting the decisions to them after appropriate counselling, made it easier for the obstetrician, resulting in less subsequent distress or guilt. These thoughts emerged in relation to discussions about the rare instances when women declined interventions for the benefit of the fetus, or where women decided on termination for minor fetal abnormalities.

### IV. Separating private from professional views

Divergent views were apparent in relation to if, or when the fetus might be regarded as a person, and the majority of participants shared their private and philosophical views when this question was raised during interviews. There was a continuum of opinions, starting with the view that the fetus became a person from conception. Some perceived the point of viability as crucial, or when the nervous system is ready to function. Others presented the view that the fetus gained more personhood the further the pregnancy advances. There were some obstetricians who stressed that the pregnant woman’s perceptions of the fetus as a person could differ quite a lot from their own, and that the pregnant woman’s opinion was what was relevant. One participant thought that the question was irrelevant because one’s own views did not impact on practice.

*‘I believe life starts from the beginning and so I don’t see anything necessary that a week of gestation makes a difference to the preceding week’* (Participant no 14)

*‘I think for me it’s from the point of viability …’* (Participant no 7)

*‘As a professional that point is when the patient decides, not me.’* (Participant no 8)

Although fetal rights were not mentioned as a topic by the interviewers, participants frequently brought the matter up themselves. All the obstetricians were unanimous in their view that a fetus only acquires legal status at birth, yet their narratives often indicated that more weight was accorded to the fetus in decision-making the further the pregnancy advanced.

*‘The common view would be that the fetus reaches viability at about 24 weeks and the welfare of the fetus starts to assume a greater weighting in the doctor’s decision making from then on.’* (Participant no 11)

*‘I think although it doesn’t have legal rights as such until it’s born in Australia, I think yes, the further along it gets, the more rights it has, in my mind.’* (Participant no 3)

While some obstetricians did not acknowledge the potential for conflict regarding maternal versus fetal health because of their clear clinical priorities, there were participants who expressed their private views on the sudden change in rights at the time of birth:

*‘I guess there’s been focus on empowering the mother, it’s the mother’s wishes, the mother’s decisions and this has a wide application within our care. And… there’s always going to be a degree of artificiality about it. I mean all of a sudden the baby’s born and suddenly it has rights whereas the day beforehand it didn’t. It’s … that doesn’t sit well with many of us but that’s just the way it is.’* (Participant no 1)

Although the obstetricians believed that the mother must take precedence over the fetus in all circumstances and felt supported in this by current legislation in the state of Victoria, they still experienced difficulty in some situations of conflicting maternal and fetal health interests.

Situations where a mother had declined interventions that would have been of benefit for the fetus, and where the mother’s decision resulted in a poor fetal outcome, were described as difficult and sometimes distressing for obstetricians. This also included terminations of pregnancies where minor fetal defects were detected through ultrasound, for example the absence of a few digits. To be able to work in obstetrics, some obstetricians thought it was important to understand and accept that the fetus has no rights.

*‘Certainly it’s been a journey for me to come to the realisation, not necessarily the acceptance but the realisation that the fetus in this jurisdiction has no rights and that we cannot act on fetal grounds without maternal consent ever.’* (Participant no 1)

*‘The natural feeling of most people, people in the street, the natural feeling is that most mothers would do whatever it takes to have a healthy outcome for the baby. And some of the most challenging examples are when women won’t … don’t do that for various reasons.’* (Participant no 1)

*‘It was a great shame [a mother declined intervention to save the fetus].. It was the equivalent of someone exsanguinating by the roadside and you weren’t able to help, this was the equivalent.’* (Participant no 8)

The obstetricians in this study expressed conflicting views about the fetus as a patient; some did see the fetus as a patient, or the pregnant woman and her fetus as ‘two patients’, while a few did not define the fetus as a patient before birth. However, there was universal agreement among the interviewed obstetricians that fetal rights were limited to those the pregnant woman assigned to the fetus, and that they could never act on fetal grounds without the pregnant woman’s consent.

## Discussion

The purpose of this study was to explore obstetricians’ experiences and views of the use of obstetric ultrasound both in relation to clinical management of complicated pregnancy, and in situations where maternal and fetal health interests conflict. The analysis resulted in four categories and one main theme that overall illuminated the challenges the obstetricians faced in balancing maternal and fetal health interests in pregnancy management. It is important to note that these interests are in fact usually aligned [22,23], and our findings should therefore be viewed in light of the specific purpose of this study.

The first category described difficult clinical situations that obstetricians sometimes face when women’s right to make autonomous decisions puts their own health and well-being at considerable risk. The results highlighted the increasing ethical conflict in balancing maternal and fetal harm and benefits with increasing advances in fetal diagnostics and treatment. In practice, ethical dilemmas occur when professionals face a conflict of obligations to the patient, and when possession of all the available factual information cannot solve the dilemma, nor can current laws [24]. In situations where maternal and fetal health interest conflict, there are several ethical theories that may aid decision making [22]. Principle-based theories including the four fundamental ethical principles of *Respect for autonomy, Beneficence, Non-maleficence,* and *Justice* are guides for action rather than rules, and a weighing up of these values is necessary for obstetricians dealing with the kinds of decision-making dilemmas described in this study [24]. The principles of beneficence and autonomy are central in the relationship between the obstetrician and the pregnant woman [25]. The principle of beneficence requires the obstetrician to protect and promote the patient’s health and to ensure that the medical benefits outweigh the burdens of treatment [26]. Respect for autonomy acknowledges the competent pregnant woman as the decision-maker for issues related to her pregnancy, including situations of maternal and fetal health conflicts [22]. It was clear in this study that the obstetricians were supportive of these principles. However, they recurrently portrayed pregnant women as having an ‘altruistic’ position in relation to their fetus. That is to say they described women in general as agreeing to a great deal of risk, and requesting interventions that could have adverse effects on their own health in order to protect the health and wellbeing of the fetus. Interestingly, others have noted that societal values and perspectives may be more sympathetic to a pregnant woman’s decision to risk her own health and wellbeing, or even life, to benefit the fetus, while decisions to prioritise one’s own health or life with adverse outcomes for the fetus may be perceived as ‘selfish’ [22]. Some argue that women’s right to make autonomous reproductive decisions is very much at stake with rapidly advancing medical technology, and further, that the focus of prenatal technologies on fetal health may lead to a reduced emphasis on maternal wellbeing [27,28]. It seems plausible that rapidly advancing medical technology in combination with societal values and expectations and the fact that women in most circumstances make choices in the best interest of the fetus [29] may over time push pregnant women to make more altruistic decisions in relation to their pregnancies. This is an important area for further study.

The second category described the obstetricians’ views of the impact of medico-technical advances on views of the fetus in relation to the pregnant woman, and of the balancing of maternal and fetal health interests. Some obstetricians provided illustrative, yet confronting examples of how the pregnant woman’s role could be marginalised, and where the focus was firmly on the fetus. These findings are consistent with existing literature which suggests that developments in medical technology have led to a change in the view of the pregnant woman and the fetus: what was previously seen as one entity (i.e. the fetus as part of the woman) has now increasingly become two distinct entities because of the possibility to view and monitor, as well as treat the fetus in utero [28,30]. By being visible to the clinician via ultrasound’s ‘window on the womb’ [27], the fetus has thus come to be considered as separate from the pregnant woman, a ‘second patient’ [22,30]. As the needs of the pregnant woman and the fetus may sometimes differ, the physician has to consider the treatment of two patients instead of one [28]. Although the obstetricians in this study presented conflicting views in relation to the fetus as a patient, they all agreed that they could never act on fetal grounds without the pregnant woman’s consent. The concept of the ‘fetus as a patient’ has been discussed and also criticized in previous literature [31-33]. While some argue that the concept avoids some of the connotations the term ‘unborn child’ can evoke, particularly in relation to ‘rights’ and the caregivers’ obligations [32], others argue that the concept of ‘the fetus as a patient’ will encourage the tendency to think of the fetus independently from the pregnant woman [33].

The third category highlighted dilemmas in relation to guiding expectant parents in making informed decisions. The obstetricians expressed concerns about complex situations where it was difficult to get relevant information across, they also thought there were situations in which it was somewhat ‘unfair’ to leave decisions to the pregnant woman or the couple. Illustrative examples of these dilemmas were the difficulties of making sense of risk probabilities regarding fetal outcomes, or when obstetricians themselves were uncertain about the significance of ultrasound findings. These situations pose conflicts for the obstetrician. Disclosing all the information has the potential to cause harm because of increased (and possibly unwarranted) worry and anxiety [9,34], yet it was evident that obstetricians in this study were very supportive of women’s autonomy and self-determination and therefore their need for full information disclosure. The purpose of the informed consent process is to enable pregnant women to exercise their autonomy [31]. The obstetrician has an important role in this process, because it is his or her obligation provide adequate, but not overwhelming amounts of medical information, tailored to each woman’s level of knowledge and education in order for her to make informed decisions. The obstetrician also needs to assist the pregnant woman or the couple in making sense of the situation and recognise the validity of their values and beliefs before action is taken in accordance with the pregnant woman’s value based preferences [31,35]. This was also emphasised by the obstetricians in this study, although it was described as a demanding task at times. For example, barriers related to culture and language [29] were mentioned as common challenges, not surprisingly in a multicultural nation such as Australia.

Consent to undergo prenatal examinations, including ultrasound, are today often taken for granted, and some women may not understand routine pregnancy ultrasound as an offer, but rather as a compulsory component of the care that they are expected to undergo during pregnancy [9,25]. Reproductive decisions are not made in a vacuum, but rather within social contexts, it is therefore likely that pregnant women will adopt and adhere to prenatal examinations that have become normalised within their social sphere [36]. Yet the obstetric ultrasound examination is at times the starting point for subsequent events and interventions that give rise to clinical dilemmas for caregivers, as well as for the woman herself and her partner [37,38]. Given the findings of this study, it would seem important that a stronger emphasis is placed on the informed consent process prior to obstetric ultrasound examinations, especially in relation to scans for screening purposes [31].

The fourth category provides a deeper insight into both the obstetricians’ private and professional views and experiences regarding the fetus as a ‘person’ and ‘patient’, and how this relates to the women’s rights of autonomy during pregnancy and childbirth. In this study, there was no consensus among obstetricians regarding if or when the fetus can be regarded as a ‘person’. Interestingly, most obstetricians indicated that they viewed the fetus as a person at some point prior to birth, with a wide spectrum presented starting from the time of conception. This lack of consensus does not seem unique to this context [28,30]. However, it has been argued that the more sophisticated the technology and imaging are, the more the fetus is considered a person, and also, that this increasing ‘personification’ may have adverse implications for women’s reproductive freedom [17]. When evaluating an ultrasound scan, the operator captures a great deal of information about the fetus, while the pregnant woman herself remains in the background. Others have described this marginalisation of the pregnant woman with metaphors including the pregnant body as an ‘empty spaceship’ for the ‘cosmonaut fetus’, a ‘fetal container’ or a ‘vessel’ [17]. It seems important to further study how these developments might influence women’s reproductive freedom and empowerment, and also what implications this potential shift of focus might have for women in different contexts.

It emerged during the interviews that those rare situations where the woman made a choice at odds with the obstetrician’s advice with adverse consequences for the fetus, were difficult and sometimes distressing for obstetricians. These findings are consistent with previous studies [29]. And although the obstetricians were unanimously supportive of women’s autonomy and felt supported by the current law, (which in Australia means that the fetus only acquires legal status at birth [39]) this acceptance of women’s autonomy in some circumstances came at a personal cost for some obstetricians, a finding also pointed out by others [29].

Pregnant women have both positive and negative rights in maternity care. By refusing interventions, women are asserting their negative right to be left alone [40], a right that should not be overridden in Australian maternity care given that the woman is able to make an informed decision [41]. Women’s positive right to request medical management can also cause dilemmas for obstetricians because the harms and benefits to mother and fetus always need to be balanced. However, obstetricians do not necessarily have to comply with a woman’s request if the suggested management is at odds with justifiable care [23]. One example is a woman’s request for caesarean section in the absence of medical indications, a situation in which the obstetrician has the right to decline to perform the procedure if there are health concerns for the mother or fetus, or if the woman does not have an understanding necessary to enable informed consent [42]. Our results are in line with these recommendations because the obstetricians saw it as their role to say ‘stop’ when non-justifiable medical management was requested.

### Strengths and limitations

A strength of this study is that participants were diverse in relation to age, gender, work experience, and work setting which likely enhances the robustness of the findings with a range of different views captured. It needs to be noted however, that as researchers we had no influence over which obstetricians fulfilling the inclusion criteria were approached and recruited via the department heads. Therefore, the possibility of some, unidentified selection bias cannot be ignored. The sensitive nature of some of the research questions under study means that we also cannot rule out the possibility that participants may not have fully disclosed the extent of their views. On the other hand, those who participated showed a great deal of interest in the topics raised and provided rich information about their views and experiences.

## Conclusions

This study suggests that medico-technical advances such as ultrasound have set the scene for increasing ethical dilemmas in obstetric practice. The obstetricians interviewed had experienced a shift in previously accepted views about what weight to give maternal versus fetal welfare. As fetal diagnostics and treatment continue to advance, how best to protect pregnant women’s right to autonomy requires careful consideration and further investigation.
